# “Position” in the Treatment of Chloroform Poisoning

**Published:** 1868-09

**Authors:** E. L. Holmes

**Affiliations:** Lecturer on Diseases of the Eye and Ear in Rush Med. Col., and Surgeon to the Chicago Charitable Eye and Ear Infirmary


ARTICLE XXXIII.

### "POSITION" IN THE TREATMENT OF CHLOROFORM POISONING.—CARBOLIC ACID IN THE TREATMENT OF CONJUNCTIVITIS.

By E. L. HOLMES, M.D., Lecturer on Diseases of the Eye and Ear in Rush Med. Col., and Surgeon to the Chicago Charitable Eye and Ear Infirmary.

The subject of anæsthetics does not fall specially within the province of your Committee on Ophthalmology; and yet, there are a few points, which I consider of some interest in connection with the administration of ether and chloroform in ophthalmic surgery.

I have observed a few facts, in operating upon the eye, which I deem of very great importance, as bearing upon the treatment of patients presenting alarming symptoms during the inhalation of chloroform. It is not my object to discuss the comparative danger in the use of chloroform or ether, nor the circumstances upon which the danger depends. It has been my fortune to observe the administration of both agents in a very large number of cases, under the care of many different and eminent surgeons. I have also administered chloroform more than 400 times myself. Till quite recently, I had never witnessed a single case in which there was any apparent danger to the patient.

Some time since, two male adult patients, to whom I administered chloroform, suddenly ceased breathing, the pulse at the wrist becoming at once imperceptible, the sound of the heart, however, being audible to the ear placed upon the chest. The removal of the chloroform, alternate pressure and relaxation of the chest, and cold water thrown upon the face, speedily aroused the action of the heart and lungs.

I may state that in administering ether to a patient 76 years of age, the respiration suddenly ceased, although the pulse, very much reduced in force, continued. The appearance of the patient was alarming, the countenance being somewhat livid, cool, and haggard in expression, and the eyes rolled upward. It required considerable exertion to restore the patient.

Every surgeon has, not unfrequently, observed that chloroform produces considerable pallor, prostration in the action of the heart, arteries, and lungs, apparently without any imminent danger. In all such cases, as well as in the two just described, the danger seems to depend entirely upon syncope. I have never witnessed a case in which there was turgidity of the vessels and redness of the face, in which there was not also a regular pulse, and a regular, though often stertorous, respiration, causing, perhaps, a peculiar heaving motion of the head. On several occasions, as I observed this tendency to syncope, although I saw no reason for alarm, I directed, experimentally, my assistants to raise the foot of the table sufficiently high to place the patient with the head downward on an inclined plane of at least 40°. I found, invariably, that the pulse at once became fuller and more frequent, and that the color returned to the face.

Subsequently, in administering chloroform to a patient at the Chicago Charitable Eye and Ear Infirmary, the breathing and pulse, almost without warning, suddenly ceased. Although the pulse and respiration had been quite good, there still had begun to be a peculiar "cold perspiration" upon the brow, and a cold, moist condition of the hands, which I attributed to the depressing influence of fear, under which the patient was laboring. I was watching the patient most carefully, thinking in this condition he should receive no more chloroform, when he ceased to breathe. His aspect was most appalling; the face and hands were cold and wet, the features pinched, muscles of the face relaxed, lids half opened, and the cornea turned upward. The foot of the table had not been raised 15 seconds, the tongue having at once been withdrawn, before the pulse reappeared at the wrist and the respiration was reëstablished. Upon restoring the patient to the horizontal position, the pulse and respiration again ceased. The elevation of the foot of the table, however, again reëstablished the action of the heart and lungs.

Some time after this occurrence, precisely the same symptoms appeared during the inhalation of chloroform. The patient was a young, strong man. In this case the pulse, for a few minutes, was growing less frequent, although the breathing continued quite strong and regular, till, without further warning, the pulse and breathing suddenly ceased. The appearance of this patient was as frightful as in the case of the other, just described. A similar mode of treatment restored, at once, the action of the heart, some seconds passing before the respiration was fully reëstablished.

I have had an opportunity, at the Infirmary, of demonstrating, experimentally, to students and physicians, more than thirty times, in cases where there was no apparent danger, and yet where there was a tendency to pallor and weakness of the pulse, that, in the position I have described, the cheeks became instantly flushed and the pulse stronger.

In administering chloroform, I always use a napkin, folded several times, upon which the anæsthetic is poured in small quantities at a time, care being taken that a free current of air can pass to the mouth under the napkin. The patients are always in a horizontal position. I watch, with great care, the condition of the pulse and respiration; and yet, it is sometimes somewhat difficult to distinguish the difference between the effects of fear and those of the chloroform.

Whatever may be the obscure causes of fatal results from the use of chloroform, I believe the danger, in by far the larger proportion of cases, depends upon a tendency to death by syncope. To overcome this tendency, it is necessary to stimulate the nervous centres. This may be done by causing a column of blood to press upon the vessels of the brain. It is not sufficient to remove the pillow from the head and place it under the hips. It is necessary that the whole body be placed upon a steep, inclined plane, to force as much blood as possible, by gravitation, into the brain. I believe this is of more importance than any of the methods usually described by writers on the subject. It should take precedence to the withdrawal of the tongue, artificial respiration, galvanism, or stimulants. This remedy can always be applied without delay, and can be followed by any others which may seem desirable.

I have dwelt upon this subject of position, because so little is said upon it, either in the best works upon anæsthetics, or in the reports of the treatment in fatal cases, as found in medical journals. We have reason to believe that very few surgeons or obstetricians have ever placed a patient in the position described, in cases which threaten to terminate fatally.

I have employed large and frequent doses of bromide of potash, as recommended by Dr. Stone, both before and after the administration of ether, as also of chloroform, to prevent nausea, but have not observed any beneficial result. It is highly desirable to possess some agent to prevent nausea and vomiting, especially in the extraction of cataract, since the vomiting may cause the expulsion of the vitreous humor, and protracted nausea, and consequent loss of appetite, may prevent union of the corneal wound, by impairing nutrition.

### CARBOLIC ACID IN THE TREATMENT OF CONJUNCTIVITIS.

The valuable properties of carbolic acid, as an application in diseases of the throat and gums, has been shown by dentists, and physicians who give special attention to diseases of the throat. The good results of its use in these diseases, induced me to try it in diseases of the conjunctiva. I have found in about a dozen cases, at the Infirmary, both of acute and, especially, of chronic inflammation, that the patients made a good recovery under its use.

It is doubtful whether it possesses any advantage over the ordinary astringents, and yet it may be a desirable agent in cases where the usual applications fail. In two cases of purulent conjunctivitis, its use between the applications of nitrate of silver, after the latter had been tried two days, seemed to rapidly overcome the excessive discharge of pus. Possibly, its antiseptic properties may aid in destroying any specific poison that may exist.

When applied, in a saturated solution, to the conjunctiva, as also to the mucous membrane of the mouth, it produces an intense burning pain, which almost invariably subsides in a few moments. It produces a thin, white pellicle on the surface where it is applied, which is soon cast off, leaving scarcely any irritation.

